# Comprehensive Transcriptome Profiling and Identification of Potential Genes Responsible for Salt Tolerance in Tall Fescue Leaves under Salinity Stress

**DOI:** 10.3390/genes9100466

**Published:** 2018-09-21

**Authors:** Erick Amombo, Xiaoning Li, Guangyang Wang, Shao An, Wei Wang, Jinmin Fu

**Affiliations:** 1 Key Laboratory of Plant Germplasm Enhancement and Specialty Agriculture and Wuhan Botanical Garden, Chinese Academy of Sciences Wuhan, Wuhan 430074, China; aeric@wbcas.cn (E.A.); lixiaoning0724@126.com (X.L.); m15506636638@163.com (G.W.); 2 The University of Chinese Academy of Sciences, 19 Yuquan Road, Beijing 100049, China; 3 The Institute of Advanced Studies in Coastal Ecology, Ludong University, Yantai 264000, China; shaoan1234567@163.com (S.A.); wangwei3456@163.com (W.W.)

**Keywords:** tall fescue, salinity stress, photosynthesis, RNA-sequencing, simple sequence repeats transcription factors

## Abstract

Soil salinity is a serious threat to plant growth and crop productivity. Tall fescue utilization in saline areas is limited by its inferior salt tolerance. Thus, a transcriptome study is a prerequisite for future research aimed at providing deeper insights into the molecular mechanisms of tall fescue salt tolerance as well as molecular breeding. Recent advances in sequencing technology offer a platform to achieve this. Here, Illumina RNA sequencing of tall fescue leaves generated a total of 144,339 raw reads. After de novo assembly, unigenes with a total length of 129,749,938 base pairs were obtained. For functional annotations, the unigenes were aligned to various databases. Further structural analyses revealed 79,352 coding DNA sequences and 13,003 microsatellites distributed across 11,277 unigenes as well as single nucleotide polymorphisms. In total, 1862 unigenes were predicted to encode for 2120 transcription factors among which most were key salt-responsive. We determined differential gene expression and distribution per sample and most genes related to salt tolerance and photosynthesis were upregulated in 48 h vs. 24 h salt treatment. Protein interaction analysis revealed a high interaction of chaperonins and Rubisco proteins in 48 h vs. 24 h salt treatment. The gene expressions were finally validated using quantitative polymerase chain reaction (qPCR), which was coherent with sequencing results.

## 1. Introduction

As a result of increased poor irrigation practices and natural soil salinization, salt stress remains a major threat to plant growth and crop productivity globally [1]. The vast area of salinized land and the heterogeneity of salinity level in the soil make it complex to study in toto [2]. Instead, it is more practical to describe the various bioprocesses that collectively confer salt tolerance to the whole plant. Tall fescue (*Festuca arundinacea* Schreb.) is a cool-season grass that is commonly used as a forage and turfgrass due to its excellent adaptability, high yield, and high aesthetic value [3]. However, its selection and utilization in salt-affected regions are limited by its inferior salt tolerance in comparison to warm-season turfgrasses [4,5]. Complex salt response mechanisms at the metabolic, physiological, structural, biochemical and molecular levels have been reported in tall fescue [6,7,8,9,10,11]. Through molecular cloning, several salt tolerant genes have been identified in tall fescue. For example, a tall fescue zinc figure gene *FaZnF* was reported to be involved in the regulation of pathways initiated by the salt-stress response [12]. In addition, an *Arabidopsis thaliana AtNHX1* gene enhanced the salt tolerance level of transformed tall fescue progenies [13], while Ma et al. [14] found out that the overexpression of *A. thaliana SOS1 + SOS2 + SOS3* genes enhanced salt tolerance in tall fescue. Recent reports on transcriptomic studies have provided insights into the mechanisms of salt tolerance in plants. For example, Wang et al. [15] uncovered novel salt responsive genes at the seedling stage of salt-tolerant Indica rice; Krishnamurthy et al. [16] identified genes that are responsible for ethylene, auxin, and abscisic acidsignaling in mangrove (*Avicennia officinalis*) growing under salt stress, while Song et al. [17] identified candidate genes that were associated with detoxification, photosynthesis, and signal transduction under long salt stress in a population of Miscanthus energy crops. Through comparative transcriptome analyses, Upadhyaya et al. [18] revealed a differential response at early and late stages of salt stress in table grapes leaves, while Sun et al. [19] observed that salt-tolerant tomato exhibited a higher expression of salicylic acid-binding protein 2 (SABP2) as well as more activation of the salt overly sensitive pathway compared to the salt-sensitive genotype. More recently, Zhu et al. [20] revealed alternative splicing under salt stress in cotton (*Gossypium davidsonii*). The transcriptome data has also enabled genome-wide studies of individual salt responsive genes as well as their functions. For example, Kumar et al. [21] reported that the ectopic expression of *OsSta2* gene in rice enhanced salt tolerance, while *AP2/EREBP* and dehydrin genes have been characterized in Cucurbitaceae species [22,23]. Also, the transcriptome of plants growing under other abiotic stresses such as cold [24,25], drought [26,27,28,29], and heat [30] have been reported. However, currently, a major constraint of understanding fully tall fescue salt tolerance is the absence of its whole genomic data. An integrated approach of combining bioinformatic tools with reported response mechanisms is imperative for unraveling crucial genome-scale information about tall fescue. In particular, the recent advancements in RNA-sequencing technology offer the high prospect for studying tall fescue transcriptome rapidly and cost-effectively, which will provide further insights into the salt response mechanism and also lay the platform for future breeding programs. 

## 2. Materials and Methods

### 2.1. Plant Growth Conditions

Tall fescue (Puregold cultivar) that we previously found to be among the most salt-tolerant was selected from a wide population through marker-assisted selection. Plant materials were collected from turfgrass germplasm center of Wuhan Botanical Garden, Chinese Academy of science. A single healthy and complete tiller (with roots and shoots) was planted in plastic pots (13 cm diameter, 11 cm deep) containing a mixture of sand and peat soil (1:1, *v*/*v*) in a greenhouse. Irrigation was performed every other day in order to maintain sufficient water supply conditions, fertilized weekly with half-strength Hoagland’s solution, and mowed to 7 cm canopy height once a week. After two months of plant establishment, tillers with uniform growth were uprooted, roots were washed with distilled water, and plants were transferred into 300 mL glass conical flasks filled with half-strength Hoagland’s solution. To prevent any algal contamination, flasks were wrapped with aluminum foil. The plants were left to acclimatize to new conditions for 7 days in growth chambers with the temperature ranging from 20 °C to 25 °C, 1000–1500 μmol photons m^−2^ s^−1^, 14 h photoperiod of natural sunlight, and 76% average relative humidity.

### 2.2. Salt Treatment

Plants were divided into two groups. One group was transferred to a fresh half-strength Hoagland’s solution (control designated CK), while the other was moved into the identical solution supplemented with 1.5% NaCl. Preliminarily, through measurement of K^+^/Na^+^ content, malondialdehyde content, electrolyte leakage, and chlorophyll content in the leaves, we observed that this salinity concentration was successful in separating salinity response of tall fescue at 0 h, 24 h, and 48 h. The materials were arranged in a randomized complete block design with multiple independent replicates. From both the control and salt treatment groups of each cultivar, leaf samples were collected for Illumina deep sequencing (two replicates) and quantitative polymerase chain reaction (qPCR) validation (three replicates), respectively. The samples were frozen immediately in liquid nitrogen and then stored at −80 °C for subsequent analysis.

### 2.3. Total RNA Extraction and Library Construction

The total RNA from the leaves of each sample was isolated using Trizol reagent (Invitrogen, Carlsbad, CA, USA) and purified using the RNeasy Plant Mini Kit (Qiagen, Valencia, CA, USA) following the manufacturer’s protocol. The RNA was analyzed using the Agilent 2100 Bioanalyzer (Agilent RNA 6000 Nano Kit Hewlett-Packard-Straß, Waldbronn, Germany). Total RNA concentration with a fragment size of 28S/18S and purity was measured using a UV spectrophotometer NanoDrop™ (Thermo Fisher Scientific, Lenexa, KS, USA), and processed by enriching the mRNA of polyA tails with magnetic beads containing Oligo(dT) followed by the fragmentation of the obtained RNA using an interrupting buffer. Random primers were used for reverse transcription, and cDNA duplex was synthesized to form double-stranded DNA. Subsequently, the synthetic double-stranded DNA was filled at the 5′ and 3′ ends followed by ligation. The ligation product was amplified by PCR (Veriti, Applied Biosystems, Thermo Fisher Scientific, Santa Fe, KS, USA) using specific primers. Briefly, the material was heat-denatured into single-stranded chains, and a single-stranded circular DNA library was cyclized with a bridging primer to obtain a single-stranded circular DNA library followed by sequencing on a computer. The sequences have been deposited in GEO NCBI with the following accession numbers: GPL23814 for Illumina HiSeq 2000 (*Festuca arundinacea*); GSM3389404 for CK; GSM3389405 for salt-24h and GSM3389406 salt-48h. 

### 2.4. Sequencing Data Filtering and De Novo Assembly

The raw data sequenced contained low-quality linker contaminants and reads with too high and unknown base content. Therefore, to ensure the reliability of results, internally developed software was used for filtering out the reads. The filtered data was designated clean reads which were candidates of de novo assembly. Trinity program version 2.8.4 [31] was used to remove PCR duplicates in order to improve the assembly efficiency of clean reads. The assembled transcripts were then clustered and unigenes were obtained. The unigenes were divided into two parts: the first were clusters, which was the result of further redundancy, beginning with CL, which denotes cluster, followed by the number of the gene family; the remaining were singletons, beginning with Unigene. 

### 2.5. Unigene Annotation and Coding DNA Sequence Forecast

The assembled unigenes were annotated by aligning them with the other seven public databases, including Pereira’s et al. [32] Non-redundant nucleotide sequences databases Nt and Nr (ftp://ftp.ncbi.nlm.nih.gov/blast/db), Tatusov et al. [33] Eukaryotic Ortholog Groups (KOG) (http://www.ncbi.nlm.nih.gov/KOG) for functional prediction. Conesa et al. [34] Basic Local Alignment Search Tool Gene Ontology (blastGO) version 2.5.0 (https://www.blast2go.com) database was used to annotate all the unigene results from the Nr database against Ashburner et al. [35] Gene Ontology (GO) (http://geneontology.org). For pathway annotation enrichment analysis, Kanehisa et al. [36] Kyoto Encyclopedia for Genes and Genomes database (KEGG) version 58 was used while Quevillon et al. [37] InterProscan database version 5.11-51.0 (http://www.ebi.ac.uk/interpro) was used to identify family members based on protein domains. Finally, Wu et al. [38] SwissProt (http://ftp.ebi.ac.uk/pub/databases/swissprot) database was used to check for better quality annotation results. Furthermore, a Transdecoder software version 3.0.1 software (https://transdecoder.github.io) recommended by Trinity was used to identify candidate coding DNA Sequences (CDS) in Unigene. Briefly, the reading frame was opened by searching for unigene families. The homologous sequence of the protein family was then searched by Blast against the SwissProt database.

### 2.6. Unigene’s Transcription Factors Coding Capacity Prediction, Simple Sequence Repeats, and Single Nucleotide Polymorphism Test

To predict salt responsive transcription factors (TFs) in tall fescue, we used Rice et al. [39] getorf database mini-size 150 (http://genome.csdb.cn/cgi-bin/emboss/help/getorf) to detect unigene’s open reading frame (ORF) and then used Mistry et al. [40] hmmsearch database version 3.0 (http://hmmer.org) to align the ORF to the TF protein domain. The aligned sequences were described according to the family of TF families listed in Zhang et al. [41] PlantfDB database version 3.0 (http://plntfdb.bio.uni-potsdam.de). Furthermore, we tested unigene simple sequence repeats (SSR)s using Thiel et al. [42] MIcroSAtellite identification tool (MISA) version 1.0 followed by Untergasser et al. [43] Primer3 software version 2.2.2 (http://bioinfo.ut.ee/primer3) to perform primer design on the detected SSRs. At the same time, we used Kim et al. [44] Hierarchical Indexing for Spliced Alignment of Transcripts (HISAT) database version 0.1.6-beta (http://ccb.jhu.edu/software/hisat) to align clean reads to the unigene, and then McKenna et al. [45] Genome Analysis Toolkit (GATK) version 3.4-0 (https://www.broadinstitute.org/gatk) was used to detect single nucleotide polymorphisms (SNPs). 

### 2.7. Unigene Expression Calculation and Differentially Expressed Gene (DEG) Detection

We calculated the differential expression of the genes between different tall fescue samples based on the gene expression and expressed as fragment per kilobase million (FPKM). A rigorous algorithm was developed for screening differentially expressed genes (DEGs) between two samples. Next, multiple hypotheses tests were made to determine the *p*-value by controlling the False Discovery Rate (FDR), as previously demonstrated by Benjamini et al. [46]. The smaller the FDR value, the more significant was the difference in expression. Therefore, in our analysis, DEGs are defined by default as FDR genes with *p* ≤ 0.001 and a fold difference of more than 2-fold. Differential gene expression comparison between libraries was done using the Poisson D method [47]. Subsequently, Langmead et al. [48] Bowtie2 software version 2.2.5 (http://bowtie-bio.sourceforge.net/Bowtie2/index.shtml) was used to align clean reads with Unigene, while Li et al. [49] RNA-Seq by Expectation-Maximization (RSEM) software version 1.2.12 (http://deweylab.biostat.wisc.edu/RSEM) was used to calculate the base of each sample according to the level of expression. Subsequently, Kumar et al. [50] time-series analysis software Mfuzz version 2.34.0 (http://mfuzz.sysbiolab.eu) was used to classify genes into multiple clusters based on similar expression profiles in order to help find functionally similar genes.

### 2.8. Functional Analysis of Differentially Expressed Genes

For functional annotation, the DEGs were classified into various GO categories. The pathway enrichment analysis was then done on the categories using the KEGG database.For protein interaction analysis, the DEGs were aligned to the von Mering’s et al. [51] Search Tool for the Retrieval of Interacting Genes/proteins (STRING) database version 10 (http://string-db.org/) using the homology with known proteins in order to investigate the interaction between DEG-encoded proteins. We then drew the first 100 relationships using Cytoscape software version 3.x. 

### 2.9. qPCR Validation 

To confirm the validity of the RNA-Sequencing (RNA-Sequencing) data, we randomly selected nine DEGs from the three libraries and their expression under salt stress was detected by qPCR analysis. Briefly, the cDNA was constructed from 3 μg of total RNA collected at the same time as the sequencing sample. Reverse transcription was performed using oligo (dT) primer following the manufacturers manual cDNA synthesis kit (Fermentas, Burlington, Ontario, Canada). Primer 5 software version 5.2.0. was used to design gene specific primers for each gene based on the target gene sequences using *EF1-1α* as an internal control. The primer use efficiencies were determined based on Robin et al. [52] and calculated using a web-based calculator (https://www.genomics.agilent.com/biocalculators/calcSlopeEfficiency.jsp). Primers that had efficiency levels ranging from 85–100% (slopes between −3.1 and −3.6 on the qPCR standard curve) were selected for expression analysis. The qPCR was done in a total volume of 20 μL, with each containing 2 μM of the forward and reverse primers, 2 μL of cDNA, and 10 μL of 2 × SYBR Green qPCR Mix (Takara, Otsu, Shiga, Japan). The thermal cycling consisted of 40 cycles of fast denaturation at 95 °C for 5 s, followed by annealing and extension at 52–55 °C for 20 s. To test the amplicon specificity, a melting curve was generated by gradually increasing the temperature to 95 °C. To determine relative fold changes for each sample, the 2^−ΔΔCT^ method was used based on normalization with the reference gene. For reliability, the PCR analysis was done thrice, and values were expressed as triplicate means. All the gene-specific primers are listed in Table S1.

## 3. Results

### 3.1. Sequencing Statistics 

A total of approximately 87.1, 85.9, and 88.4 million raw reads were obtained from CK, salt-24h, and salt-48h, respectively. After filtering out contaminants, approximately 66.6, 65.9, and 65.8 million clean reads were obtained in CK, salt-24h, and salt-48h, respectively. Within the clean reads, a total of 96.41% and 96.31% bases in salt-24 and salt-48h had mass values greater than 20%, while 89.1% and 89% had bases with mass values greater than 30%, see Table 1.

### 3.2. De Novo Assembly and Unigene Annotation

Due to the lack of tall fescue genome, to improve assembly efficiency, the clean libraries were de novo-assembled into one reference transcriptome with the program Trinity. The assembler generated a mean total of 150,957 (CK), 152,599, (salt-24h), and 152,317 (salt-48h) reads with GC contents of 50.2%, 50.4%, and 50%, respectively. Of the three libraries, salt-48h had the highest total length of 99,403,369, while CK had the least. The mean length ranged from 646 to 652 nt across the three libraries, see Table 2a. Subsequently, a cluster deduplication was performed to get the final unigene for a subsequent analysis designated All-unigene. As a result, a total of 144,339 All-unigenes were generated with a mean length of 898 and GC content of 49.49%, see Table 2b. 

**Figure 1:**
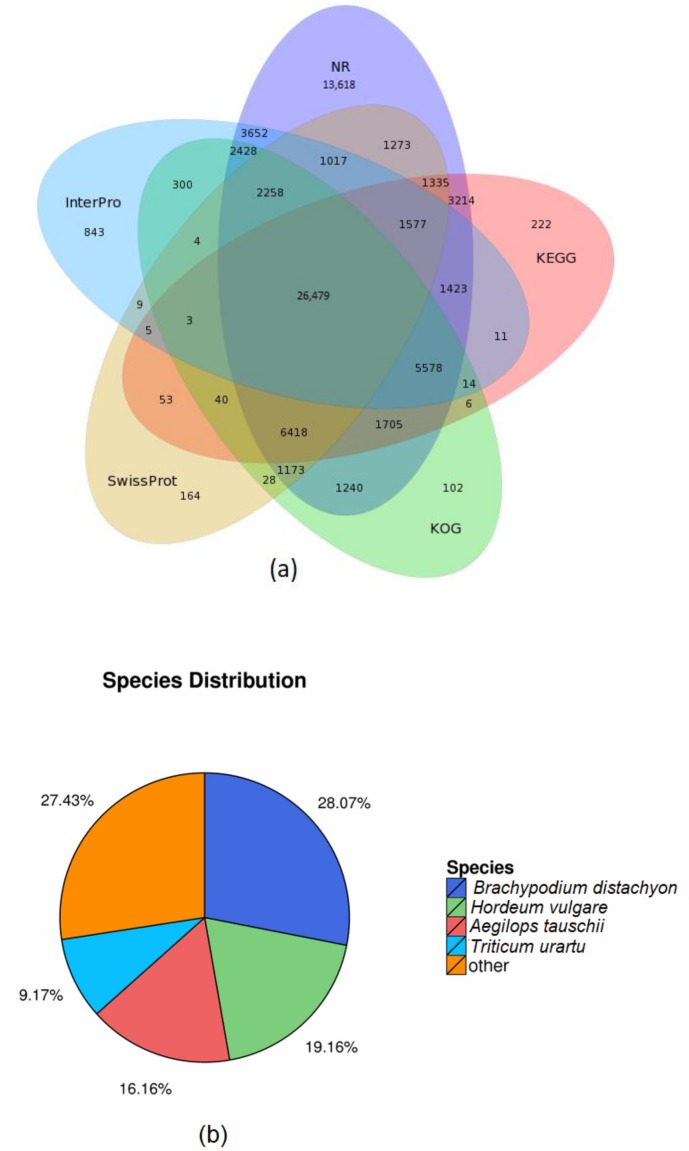
Graphical representation of unigenes based on (**a**) Venn diagram depicting the overlap in various databases. The number outside the circle denotes the total number of unigenes in each database. The number within one circle denotes the overlapped unigenes, respectively. (**b**) Species distribution showing the proportions of different species associated with the unigene annotations according to the results of the NR annotations.

**Figure 2:**
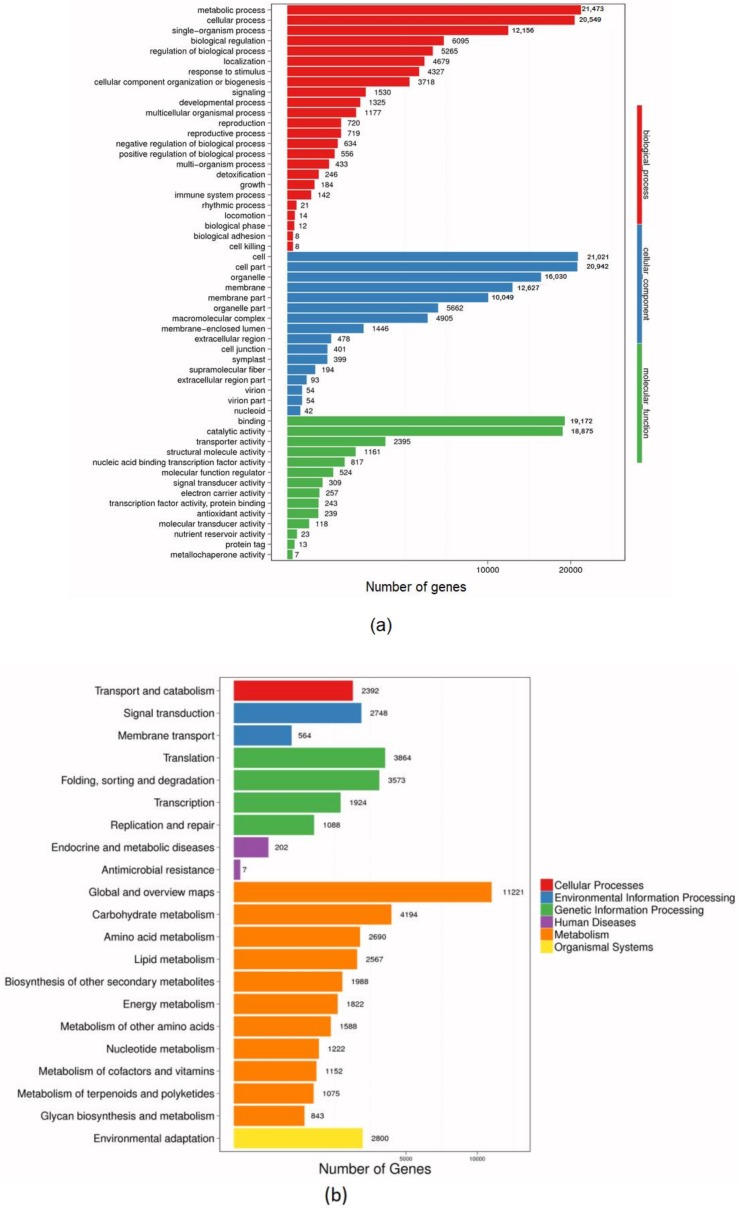

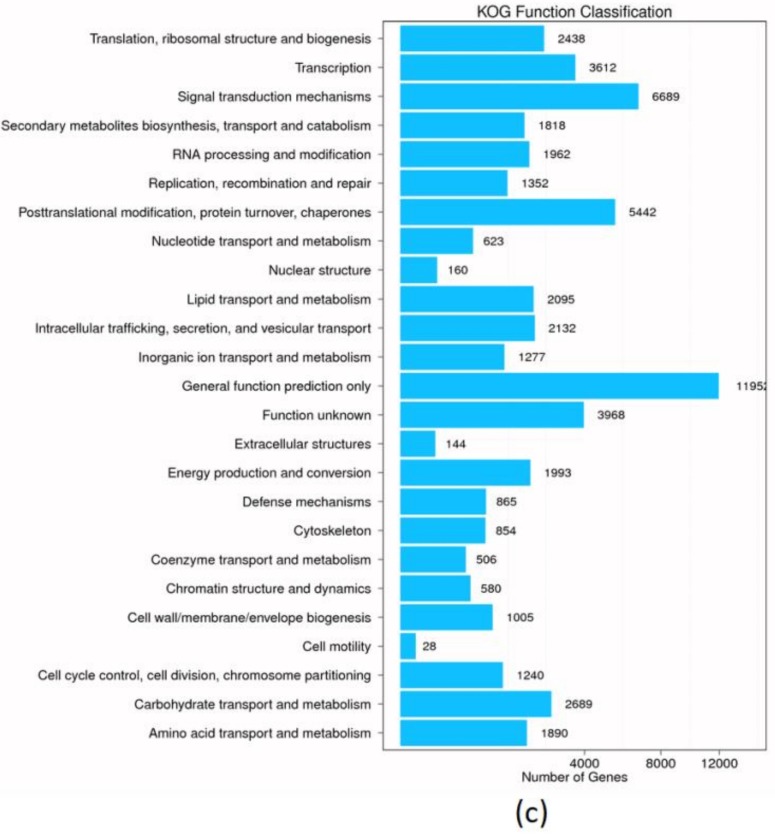
Graphical representation of unigene distribution based on (**a**) Gene Ontology (GO) classification and (**b**) Kyoto Encyclopedia of Genes and Genomes (KEGG). Genes are divided into six branches according to the participating KEGG metabolic pathways: cellular processes, environmental information processing, genetic information processing, human diseases, metabolism, organisms, medicines drug development. (**c**) Eukaryotic Ortholog Groups (KOG) annotation of putative proteins. All 47,776 putative proteins showing significant homology to those in the KOG database were functionally classified into 25 groups. The *X*-axis represents the corresponding unigene number, and the *Y*-axis represents the functional classification.

A total of 74,388 (51.5%), 62,387 (43.2%), 41,836 (28.9%), 48,083 (33.3%), 47,776 (33.1%), 45,601 (31.6%), and 40,017 (27.7%) reads were aligned in Nr, Nt, SwissProt, KEGG, KOG, Interproscan, and GO databases, respectively, see Table S2. Furthermore, 17,928 Unigenes were found in all seven databases; while 83,213 unigenes were not found in more than one of the seven databases, see Figure 1a and Table 3. Most of the transcripts had remarkable sequence similarity with *Branchypodium distachyon*, *Hordeum vulgare*, *Aegilops tauschii*, *Triticum urartu*, and others, which each of the above sharing 28.07%, 19.16%, 16.16%, 9.17%, and 27.43% of the total number of transcripts, respectively, see Figure 2b.

In total, 40,017 unigenes were classified into 54 GO functional groups and were divided into three major categories, i.e., *biological process*, *cellular component*, and *molecular function*. Under *biological process*, *metabolic process* constituted the largest sub-category with 21,347 genes. Other categories with a high number of genes included *cellular process* (20,549), *single organism process* (12,156), and *biological regulation* (6095). The least number of genes were observed in *cell killing* (8), *biological adhesion* (8), *biological phase* (12), and *locomotion* 14. Within *cellular component*, *cell* and *cell part* had the most unigenes at 21,021 and 20,942, respectively. *Nucleod*, with 42 unigenes, constituted the least number of genes. Under *molecular function*, *binding* and *catalytic activity* were the most represented sub-categories with 19,172 and 18,875 unigenes, respectively, while *metallochaperone activity*, with only seven unigenes, constituted the least represented group, see Figure 2a. We further analyzed pathway enrichment of All-unigene using the KEGG database. As a result, 48,083 unigenes were assigned to 21 pathways that were classified into six major categories, i.e., *cellular process*, *environmental information processing*, *genetic information processing*, *human diseases metabolism*, and *organism systems*. *Metabolism category* was the most enriched [11] under which the *global overview maps* sub-category was the most represented followed by *carbohydrate metabolism*. Under *genetic information processing* category, *translation* sub-category was the most enriched pathway. Despite having only one sub-category, *environmental adaptation* was highly enriched with 2800 unigenes, see Figure 2b. At the same time, 47,776 unigenes from the KOG database were divided into 25 functional groups. Among the categories, *general function prediction only*, with 11,952 genes, constituted the largest group, while *cell motility*, with only 28 genes, constituted the smallest group. Other groups with notably many unigenes included *signal transduction mechanism* (6689 genes); *post-translational modification*, *protein turn over, chaperones* (5442 genes); *transcription* (3968); *transcription factors* (3612); and *carbohydrate transport and metabolism* (2689), Figure 2c.

### 3.3. Unigene’s Transcription Factors Coding Capacity Prediction and Unigene’s Coding DNA Sequence Forecast

**Figure 3:**
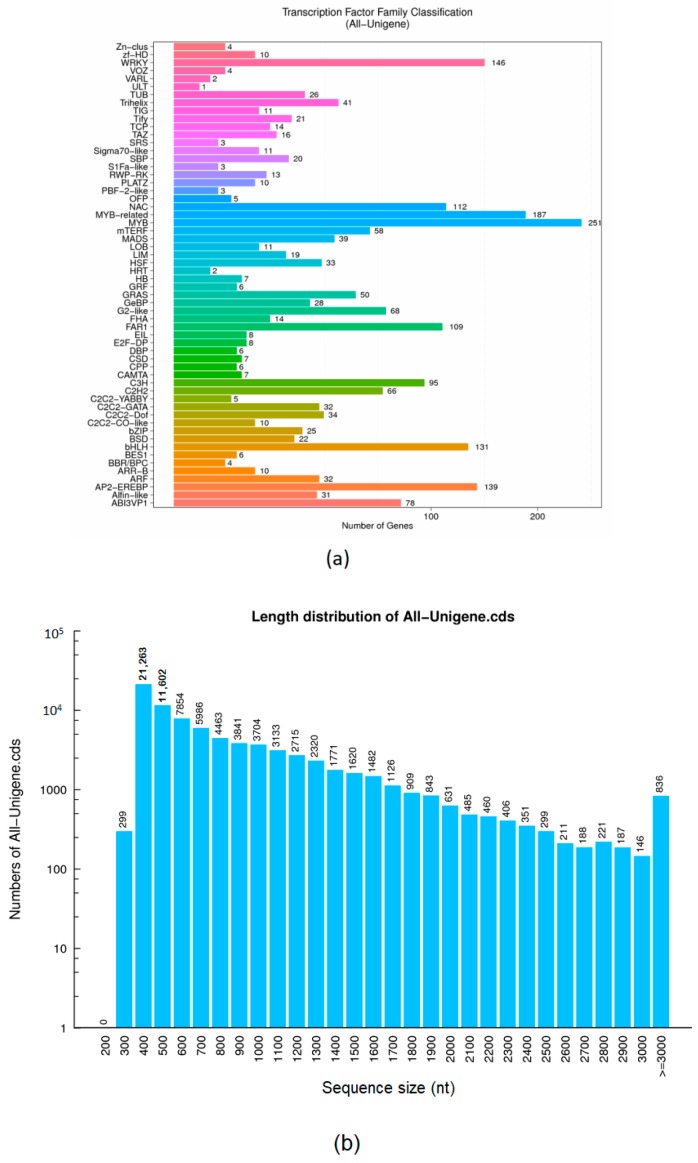
Bar plots showing the distribution of (**a**) Transcription factors. The *X*-axis represents the corresponding number of unigenes, and the *Y*-axis represents the transcription factor family classification. (**b**) Coding DNA sequence (CDS) length. The *X*-axis represents the length of the CDS, and the *Y*-axis represents the corresponding number of CDSs.

The assembled transcriptome of salt-stressed tall fescue leaves showed that 2120 completely unique TFs from 59 different families were differentially expressed during early salinity stress. In particular, the most expressed TF constituted key families that are salt-responsive, i.e., transcriptional activator Myb (MYB) (251), MYB-related (187), NAC domain-containing proteins (NAC) (112), WRKY transcription factors (WRKY) (146), APETALA2 and ethylene-responsive element binding proteins (AP2-EREBP) (139), and Basic helix-loop-helix (BHLH) (131) categories (see Figure 3a and Table S3). TransDecoder software identified a total of 79,352 sequences with a total length of 63,662,448. The length of the sequences ranged from 297–12,375, with a mean length of 297 and GC content of 53.95. A sequence size of 400 nt constituted the most All-unigene-CDS (see Figure 3b and Table S4). 

### 3.4. Unigene’s SSR and SNPs Test

**Figure 4:**
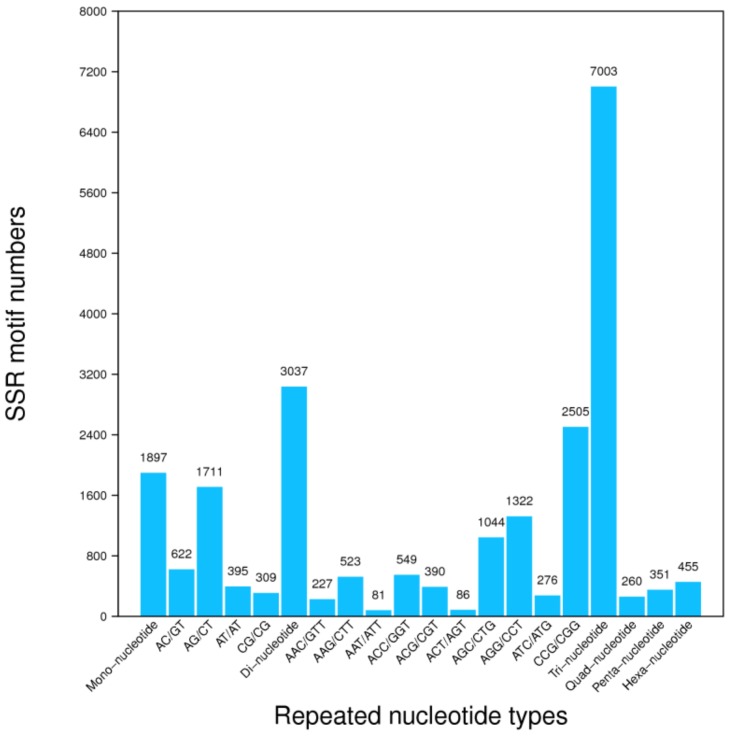
Bar plot of simple sequence repeats (SSR) distribution. The *X*-axis represents the SSR type, and the *Y*-axis represents the corresponding number of SSRs.

A total of 22,328 SSRs were identified in contig sequences. The SSRs included both single and compound SSRs. Among the compound SSRs, tri-nucleotide constituted the largest group followed by di-nucleotides. Mononucleotides consisted of 1897 motif numbers, Figure 4. Based on the assembly results, we designed primers for each SSR and defined their length characteristics, Table S5. Furthermore, based on the assembly results, we detected the SNP of each sample. Among the SNPs, 209,859, 187,300, and 189,920 had A-G and C-T mutations (i.e., they contained substitution between purines and purines, or the substitution between pyrimidines and pyrimidines) in CK, salt-24h, and salt-48h, respectively, while 117,382, 105,488, and 106,858 had A-C, A-T, C-G, and G-T variants of SNPs (i.e., had substitutions between purines and pyrimidines) in CK, salt-24h, and salt-48h regimes, respectively, Table 4. 

### 3.5. Differential Gene Expression and Distribution in Samples

According to the assembly results, we mapped the clean reads of each sample to Unigene and then calculated the gene expression level of each sample. A mean total of 50,987,376, 51,527,965, and 50,782,868 reads were mapped in CK, salt-24h, and salt-48h, respectively. Among them, 21,680,100, 21,538,592, and 21,848,553 unique reads were mapped, Table 5.

**Figure 5:**
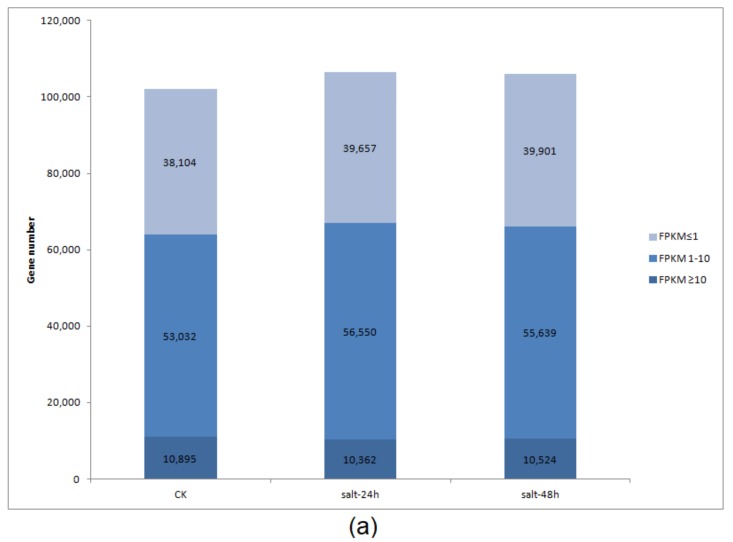

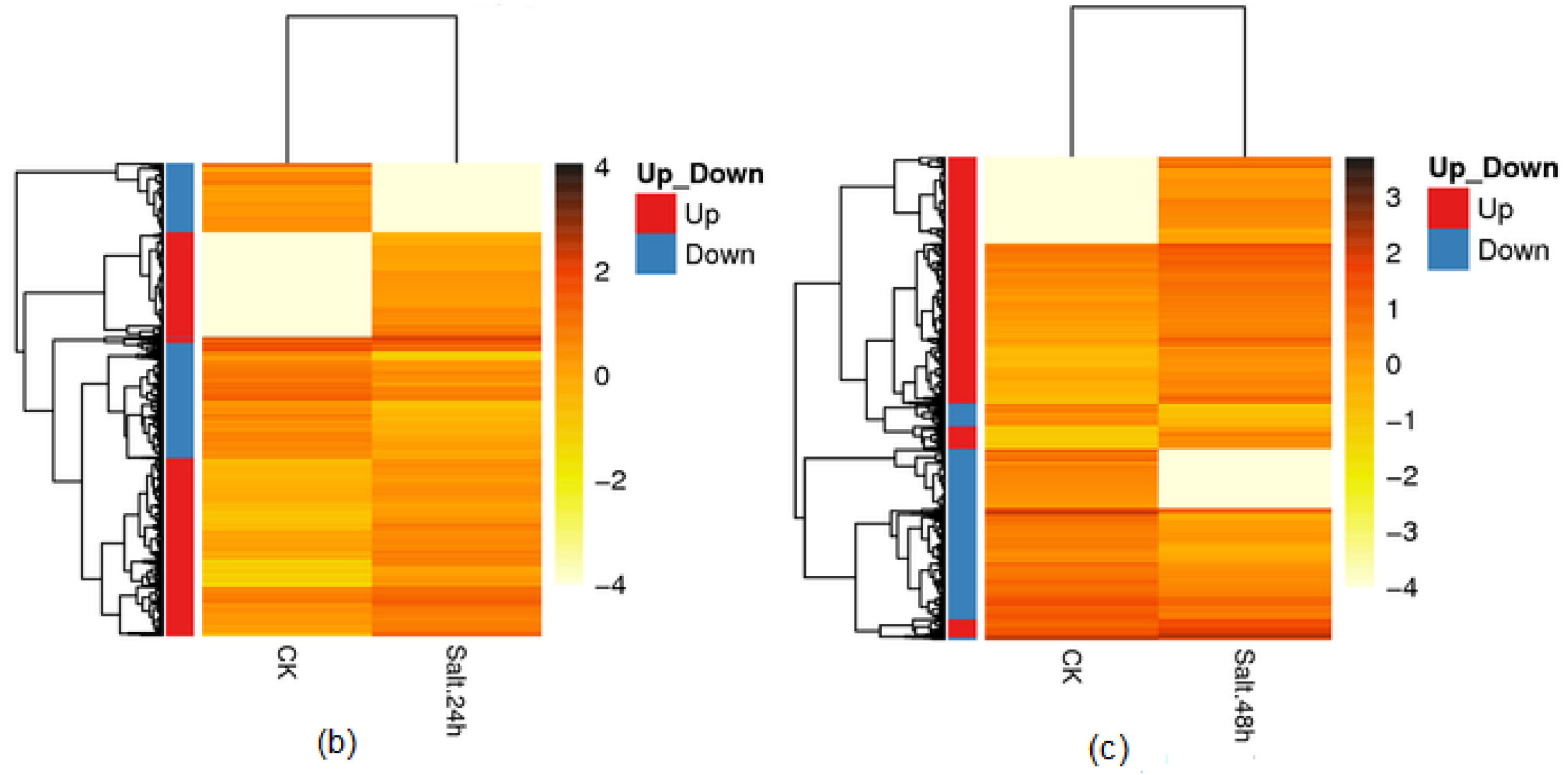
Plots of differentially expressed genes (DEGs) based on (**a**) gene abundance in samples as a function of expression levels in different fragment per kilobase million (FPKM) intervals. The X-axis represents the sample name, the Y-axis represents the number of genes, and the shade of color represents the level of different expression levels: FPKM ≤ 1 is when a gene has a very low expression level, FPKM is between 1 and 10 (when genes have low expression levels), and FPKM ≥ 10 are genes with high expression levels. (**b**,**c**) A heat map showing differential gene expression; the X-axis represents different samples and the Y-axis represents the differential gene. The darker the color, the higher the expression, while the lighter the color, the lower the expression.

Furthermore, an average of 102,031, 106,569, 106, 065 genes were differentially expressed in the CK, salt-24h, and salt-48h, respectively, at FPKM ≤ 1, FPKM 1–10, and FPKM ≥ 10, Figure 5 and Table S6. Mfuzz software revealed that all the DEGs fell into 12 clusters. With 25,854 DEGs, cluster 12 was the largest, followed by cluster 11 (14,100), while cluster four had only 5113 DEGs, Table S7. 

**Figure 6:**
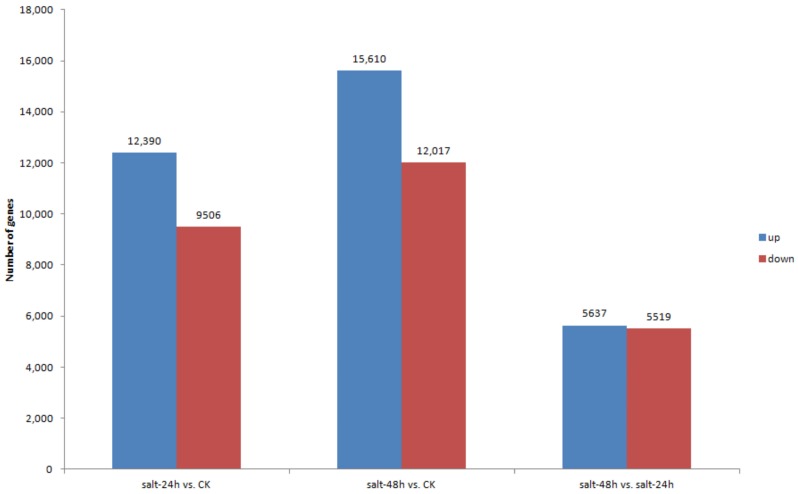
DEGs quantity chart. The X-axis represents each set of differential alignment schemes, and the Y-axis represents the corresponding number of DEGs. Blue represents the number of upregulated DEGs and red represents downregulated DEGs.

Subsequently, based on the results of the gene expression levels in each sample, we used the Poisson D algorithm to compare DEG expression levels between samples. At *p* ≤ 0.001 in salt-24h vs. CK, 12,930 DEGs were upregulated while 9506 were downregulated. In salt-48h vs. CK, 15,610 were upregulated and 12,017 were downregulated, indicating an increase; while in salt-48h vs. salt-24h regime, 5637 DEGs were upregulated while 5519 were downregulated, indicating that more transcripts were uniquely expressed with an increase in salt treatment time, Figure 6 and Table S8. Among the upregulated genes in salt-48h vs. salt-24h were *NAC67* (Unigene16967_All), *NAC48* (CL5587.Contig1_All), *NAC021* (CL19340.Contig2_All), *WRKY20* (CL5384.Contig1_All), *WRKY46* (CL12389.Contig1_All), and *ERF1* (CL914.Contig6_All) among others. Also upregulated were regulators of antioxidant enzyme activities such as *CATALASE 1* (*CAT1*) (CL342.Contig3_All) and *CAB1* (CL1733.Contig11_All). Others are photosynthesis-related genes such as *CLH2* (CL13725.Contig1_All), *WHAB1.6* (CL1733.Contig12_All), and *VAR3* (Unigene12326_All), Table 6. 

### 3.6. Functional Analysis of Differentially Expressed Genes

The three major functional categories were classified into 53 functional groups. Generally, the GO annotation trend was not so different among the three libraries, despite some differences in the number of annotated genes. For example, compared to the control, at 24 h of salt treatment, under *molecular function*, most genes were annotated to *catalytic activity* (4021) and *binding* (3574). Under *cell component*, *cell* (3972) and *cell part* (3951) constituted the largest group. Under *biological process, metabolic process* (4345) and *cellular process* (4118) constituted the largest groups. Among the highly annotated genes, a majority were upregulated. At 48 h the number of annotated genes increased remarkably. Under *molecular function*, *catalytic activity* and *binding* still contained the most genes; however, the number of genes was remarkably higher compared to 24 h. The same applied to other categories. Pathway enrichment analysis on the three major functional categories showed that *metabolic pathways* were the most enriched, with over 1000 genes in 24 h compared to control, and then followed by *biosynthesis of secondary metabolites*. Differently, at 48 h, *biosynthesis of secondary metabolites* was the most enriched followed by *plant hormone signal transduction*, Figure S1.

### 3.7. Differential Protein Interaction Analysis 

**Figure 7:**
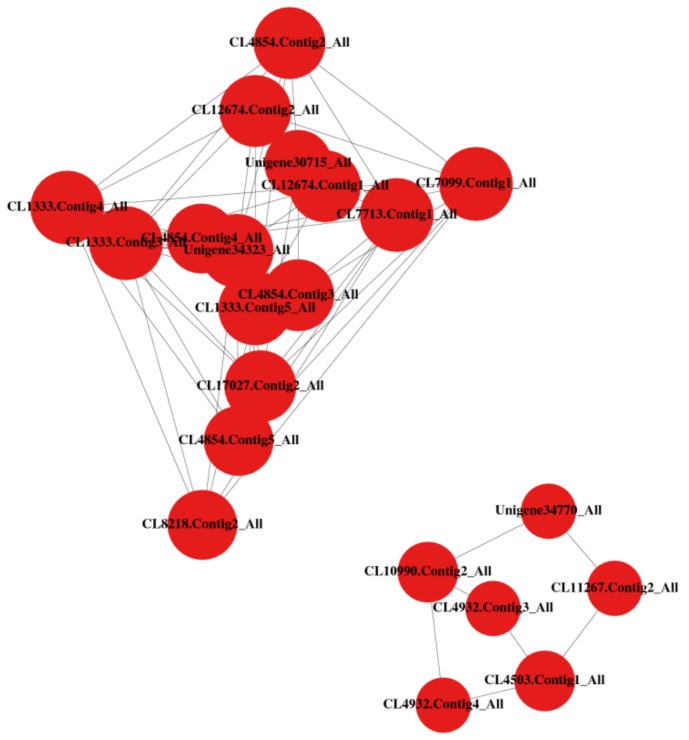
A representative of the most upregulated and highest protein interaction data in salt-48h vs. salt-24h. The larger the circle, the denser the relationship. The CL at the beginning of a name denotes a cluster and is followed by the number of the unigene family.

We used the STRING (http://string-db.org) protein interaction database to perform a protein interaction analysis for each group of DEGs. With the highest interaction and upregulation were Rubisco large subunit-binding protein subunit (CL1333.Contig4_All, Unigene34323_All, CL7713.Contig1_All) and chaperonins, such as CPN60-ß (CL7099.Contig1,2&3_All), CPN21(CL17027.Contig 2,3 &4_All), and groS-A (CL8218.Contig2_All), Figure 7 and Figure S2. 

### 3.8. qPCR Validation

**Figure 8:**
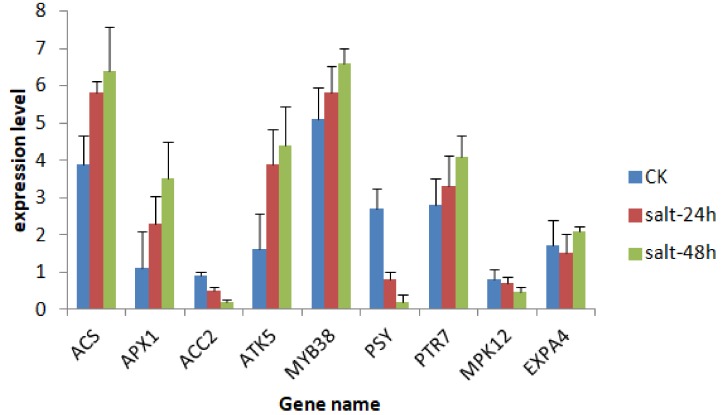
Bar plot showing the expression analysis of randomly selected RNA-Seq genes by quantitative polymerase chain reaction (qPCR). *EF1-a* gene was used as the reference gene for normalization of gene-expression data. Values are the average of three independent replicates. <1 is downregulation while >1 is upregulation.

All the randomly selected genes were detected via qPCR. *ACS*, *APX1 ATK5 MYB38 PTR7*, and *EXPA4* were highly upregulated, especially at 48 h compared to 24 h, while *ACC2*, *MPK12*, and *PSY* were downregulated, which corresponds to RNA-seq data, thus indicating its validity, Figure 8.

## 4. Discussion

Like other mesophytes, the harmful effects of salinity on tall fescue are attributed to Na^+^ and Cl^−^ -induced ion toxicity, physiological drought, and nutrient imbalances [66]. Hence, the transcript response in tall fescue is putatively aimed at ameliorating/preventing the three limiting factors via triggering a broad array of activities that optimize plant performance under saline conditions. 

GO, KEGG, and KOG are vital databases of determining potential gene functions. The differential annotation of genes in salt-24h and salt-48h compared to CK, and their enrichment in *binding*, *cell and metabolic pathways* in the KEGG database implied that salt stress duration played a role in triggering a remarkable difference in not only the transcript level but also in the gene function, hence, sampling time is crucial in assessing salt-responsive gene function in tall fescue. Previously, under water stress, Talukder [67] observed that *proteolysis*, *nucleus,* as well as *ATP binding,* contained the most genes in *biological process*, *cellular part*, and *molecular function* categories, respectively. Differently, in our study, *metabolic process*, *cell*, and *binding* had the largest unigenes in *biological process, cellular part*, and *molecular function*, respectively. This remarkable difference in GO annotations of same species might be attributed to the nature of stress differences. However, consistent with our study, under heat [68] and lead stress [69] *metabolic processes* and *binding* constituted the largest group of unigenes in biological and molecular function categories, respectively. This was also similar in Tibetan barley growing under low nitrogen conditions [70]. This observation highlighted the possible overwhelming importance of genes annotated to *metabolic process* and *binding* sub-categories in response to various environmental conditions.

Most of the predicted TFs such as WRKY, BHLH, MYB, NAC, and ERF have clear roles in growth and developmental programs in plants under salt stress. Here, we discuss TFs that were upregulated in salt-48h vs. salt-24h after salt treatment. For instance, rice plants overexpressing NAC67 accumulated higher biomass and improved salt tolerance [65]. NAC021 is a transcriptional activator that plays an important role in growth and salt tolerance [53,54]. Lower membrane damage and improved salt tolerance were observed in transgenic alfalfa, overexpressing WRKY20 [56]. The expression of a WRKY transcription factor WRKY46 is rapidly induced by salt stress and regulated a set of genes involved in cellular osmoprotection and redox homeostasis [57]. Also upregulated are key genes responsible for various metabolic processes such as carbohydrate metabolic process during glycolysis like *PFK6* [61] and antioxidant activities. Plants have developed protective mechanisms to eliminate or reduce reactive oxygen species (ROS) and other oxidants during salt stress and the enzymatic antioxidant system is one of the protective mechanisms. The remarkable upregulation of *CAT1* genes by salt stress at 48 h vs. 24 h in this study is consistent with the previous report that their activity and transcript levels were increased under salt stress [59,60]. Plants’ photosynthetic capacity under salt stress can be an important determinant of tolerance level [71]. Here, some genes associated with photosynthesis were differentially expressed and upregulated in 48 vs. 24 following salt treatment. For example, *GAPC* maintains photosynthesis under salt stress in *Thellungiella halophila* [58]. A zinc finger gene *VAR3* is part of a protein complex required for normal chloroplast and palisade cell development [62]. Chlorophyll a-b binding genes *WHAB1.6* and *CAB1* encode the major chlorophyll polypeptide, which functions as a light-harvesting complex that captures and delivers excitation energy to photosystems [63,64]. Salt stress signaling genes were also upregulated in 48 h vs. 24 h of salt treatment. For instance, *ERF1* regulates ROS-dependent signaling during the initial response to salt stress in rice [55]. The temporal differential expression of the genes and their upregulation in 48 h vs. 24 h indicates that prolonged stress generated a unique gene expression pattern, which represents regulatory transcript responses that cannot be predicted from the early response. In addition, an overlap of transcripts in between 48 h salt and 24 h salt suggests that some transcripts were not temporal, and their expression was an important early and late response mechanism to salt stress.

Furthermore, the CDS forecast enabled us to predict the potentially different protein outputs at different transcript levels. ‘All Unigenes’ at 400 nt length were most enriched and might contain a majority of potential candidates to be converted into amino acids by the ribosomal translation machinery. Proteins usually perform their functions after they have been combined into complexes through interactions. Thus, to increase the statistical power we mapped the DEGs directly to candidate protein interaction networks. As a result, we see an upregulation and high interaction level of Rubisco and chaperonin proteins. Chaperonins are a class of molecular chaperones that mediate the folding of non-native polypeptides with concomitant ATP hydrolysis. Salt-responsive chaperonins such as CHAPERONINS 10, 21, and 60-ß were found to play important roles in plants response to salt stress [72,73]. Also, Rubisco is an enzyme that is involved in the first major step of carbon fixation [74,75]. These results suggest that high interaction of chaperonin-related and Rubisco genes as well and their upregulation in 48h-salt vs. 24h-salt might have played important role in salt responsive protein folding as well as modulation of photosynthesis in tall fescue leaves. Also, it is possible that the upregulation of Rubisco in tall fescue reflects the increase in photorespiration during exposure to salt stress. 

Primarily, transcriptome data is a vital tool for the fast and cost-effective creation of molecular markers. In particular, SNPs and SSR markers are vital tools for genomic and plant breeding studies. Their detection and successful primer design indicate that our tall fescue transcriptome data offered a rich genomic resource to develop a large number of molecular markers with promising potential to be used in marker-assisted selection for plant breeding, especially in the absence of full genomic sequencing information of tall fescue. In addition, the high abundance of SNP markers may be useful for molecular research of tall fescue in the case of the absence of SSR markers. 

## 5. Conclusions

In summary, to understand a tall fescue transcriptome under salt stress, RNA extracted from three tall fescue (‘Puregold’ cultivar) leaf libraries (CK, salt-24h, and salt-48h) were sequenced. After assembly, unigenes and their various characteristics were obtained. The unigenes were subsequently aligned to seven databases for functional annotations, which grouped them into various categories including those that may play important roles in salt tolerance. In addition, CDSs were detected as well as SSRs and SNPs that were distributed among the unigenes. Also predicted were key TFs, most of which belong to salt-tolerant families. Finally, DEGs per sample were analyzed and annotated to various functional groups using the GO and KEGG databases. This is the first RNA-seq study to profile tall fescue transcriptome under salt stress conditions.

## Figures and Tables

**Table 1:** Quality statistics of filtered reads.

Sample	Total Raw Reads (Mb)	Total Clean Reads (Mb)	Total Clean Bases (Gb)	Clean Reads Q20 (%)	Clean Reads Q30 (%)	Clean Reads Ratio (%)
CK	87.16	66.65	6.67	96.48	89.35	76.47
Salt-24h	85.915	65.965	6.595	96.41	89.175	76.8
Salt-48h	88.405	65.825	6.585	96.315	89.01	74.47

CK: Control; Total Raw Reads (Mb): The number of reads before filtering; Total Clean Reads (Mb): Filtered reads Total Clean Bases (Gb): Total number of bases after filtration; Clean Reads Q20 (%): The percentage of the number of bases with mass values greater than 20% in the filtered reads; Clean Reads Q30 (%): The percentage of the number of bases with a mass value greater than 30% in the filtered reads; Clean Reads Ratio (%): The proportion of filtered reads.

**Table 2:** (**a**) Trinity statistics. (**b**) Unigenes statistics.

Sample	Total Number	Total Length	Mean Length	N50	N70	N90	GC (%)
CK (a)	150,967	98,232,067	650	1034	598	261	50.23
Salt-24h (a)	152,598.5	98,743,637	646	1024	592.5	261	50.395
Salt-48h (a)	152,316.5	99,403,369	651.5	1040	603	261.5	50.035
CK (b)	86,828	63,981,634	736	1147	679	301	49.69
Salt-24 (b)	88,639.5	65,201,778	734.5	1137.5	677	303	49.875
Salt-48 (b)	87,520.5	64,836,088	740	1152	689	303	49.5
All-Unigene (b)	144,339	1.3 × 10^8^	898	1443	912	367	49.49

Total Number: number of reads per sample; Total Length: reads length of each sample; Mean Length: average length of reads; N50: used to measure the continuity of the assembly, the larger the value, the better the assembly effect; N50, N70 and N90: the percentage of the total length which is the last accumulated value after sorting by the transcript length from big to small and accumulating all transcriptions one by one; GC (%): Ratio of bases G and C.

**Table 3:** Table of alignment results for the seven databases.

Values	Total	Nr	Nt	SwissProt	KEGG	KOG	Interpro	GO	Intersection	Overall
Number	144,339	74,388	62,387	41,836	48,083	47,776	45,601	40,017	17,928	83,213
Percentage	100%	51.54%	43.22%	28.98%	33.31%	33.10%	31.59%	27.72%	12.42%	57.65%

Intersection: The total number and proportion of unigenes in all database notes in the seven databases; Overall: The total number and proportion of unigenes in any one of the seven databases. NT is NCBI official nucleic acid sequence database, NR is the official protein sequence database, with comprehensive, non-redundant features. We divided the NT and NR databases in order to annotate the unigene sequence to the corresponding points to get corresponding functional results. The Venn diagram in Figure 1 shows the sharing of unigenes in different databases. KEGG: Kyoto Encyclopedia for Genes and Genomes; KOG: Eukaryotic Ortholog Groups; GO: Gene Ontology.

**Table 4:** Integrated results of each sample single nucleotide polymorphism (SNP).

Sample	A–G	C–T	Transition	A–C	A–T	C–G	G–T	Transversion	Total
CK	105,203	104,656	209,859	28,471	20,647	39,544	28,720	117,382	327,241
Salt-24h	93,988	93,312.5	187,300.5	25,432	18,548.5	35,876	25,632	105,488.5	292,789
Salt-48h	95,195	94,725.5	189,920.5	25,844.5	19,188.5	35,741.5	26,083.5	106,858	296,778.5

A–G: Number of SNPs with A–G mutations (including A to G, G to A); C–T: Number of SNPs with C–T mutations; Transition: The number of SNPs with A–G and C–T mutations, the substitution between purines and purines, or the substitution between pyrimidines and pyrimidines; A–C: Number of SNPs with A–C variation; A–T: Number of SNPs with A-T variation; C–G: Number of SNPs with C–G mutation; G–T: Number of SNPs with G–T mutation; Transversion: A–C, A–T, C–G, and G–T variants of SNPs, substitutions between purines and pyrimidines; Total: The total number of all variant types.

**Table 5:** The statistics of the differentially expressed genes.

Sample	Total Bases	Total Reads	Total Mapped Reads	Unique Mapped Reads
CK	6,665,213,000	66,652,130	50,987,376	21,680,100
Salt-24h	6,596,346,200	65,963,462	51,527,965	21,538,592
Salt-48h	6,582,573,600	65,825,736	50,782,868	21,848,553

Sample: sample name; Total Bases: The number of bases for the sample; Total Reads: Number of reads for samples; Total Mapped Reads: Reads of all referenced sequences; Unique Mapped Reads: The number of reads aligned to a unique position in the reference sequence.

**Table 6:** Some of the selected salt-responsive and photosynthesis-related genes that were upregulated at 48 h of salt treatment compared to 24 h.

Unigene	log_2_ Fold Change (Salt-48h/Salt-24h)	Gene name	Function	Reference
CL19340.Contig2_All	1.048	NAC021	Salt tolerance	[53,54]
CL914.Contig6_All	8.247	ERF1	ROS signaling	[55]
CL5384.Contig1_All	2.098	WRKY20	Salt tolerance	[56]
CL12389.Contig1_All	2.228	WRKY46	Osmotic stress response	[57]
CL8335.Contig1_All	1.3	GAPC	Photosystem repair and salt tolerance	[58]
CL342.Contig3_All	1.1	CAT1	Response to oxidative stress	[59]
CL16806.Contig2_All	1.7	APX2	Response to oxidative stress	[60]
Unigene27613_All	1.5	PFK6	Fructose 6-phosphate metabolic process;	[61]
Unigene12326_All	2.3	VAR3	Chloroplast development	[62]
CL1733.Contig12_All	1.4	WHAB1.6	Photosynthesis, light harvesting in photosystem I	[63]
CL1733.Contig11_All	1.3	CAB1	Photosynthesis, light harvesting in photosystem I	[64]
Unigene16967_All	2.362	NAC67	Salt tolerance	[65]

The CL at the beginning of a unigene name denotes a cluster and is followed by the number of the gene family; ROS is reactive oxygen species.

## Supplementary Materials

The following are available online at http://www.mdpi.com/2073-4425/9/10/466/s1, Figure S1: Functional annotation of DEG, Figure S2: Protein interaction network, Table S1: qPCR primers, Table S2: Functional database annotations, Table S3: Transcription factors, Table S4: Coding DNA sequence, Table S5: SSR primer sequence, Table S6: Differentially expressed genes, Table S7: DEG clusters, Table S8: Comparative differential gene expression.
